# Implementing a new model of substance use care in British Columbia: healthcare staff experiences from the road to recovery initiative

**DOI:** 10.1186/s13722-026-00705-y

**Published:** 2026-08-06

**Authors:** Ava Abramowich, Piper Dickhout, Seonaid Nolan, Cameron R. Eekhoudt, Sidney Roberge, Travis De Wolfe, Erika Mundel, Renée M. Janssen, Andrea Ryan, Cheyenne Johnson, Laurel Lemchuk-Favel, Harmony Johnson, Jeanette M. Bowles, Brittany B. Dennis

**Affiliations:** 1 British Columbia Centre on Substance Use, 400-1045 Howe St., Vancouver, BC V6Z 2A9 Canada; 2 Faculty of Science, University of Southern California, Zonal Avenue, Biggy Street, Los Angeles, CA 90033 USA; 3 Division of Social Medicine, Department of Medicine, University of British Columbia, 327-2194 Health Sciences Mall, Vancouver, BC V9T 1Z3 Canada; 4 Interdepartmental Division of Addiction, Providence Health Care, 1081 Burrard St., Vancouver, BC V6Z 1Y6 Canada; 5 Department of Medicine, University of British Columbia, 327-2194, Health Sciences Mall, Vancouver, BC V9T 1Z3 Canada; 6 Indigenous Wellness and Reconciliation Team, Providence Health Care, 1081 Burrard St., Vancouver, BC V6Z 1Y6 Canada

**Keywords:** Addiction medicine, Substance use, Health service evaluation, Withdrawal management, Detox, Healthcare staff, Job satisfaction, Opioid use disorder, Overdose, Quality improvement, Mixed methods

## Abstract

**Background:**

To address the increasingly potent unregulated drug supply and the complex withdrawal syndromes that accompany it, a hospital-based withdrawal management unit was launched in September 2023 as part of Vancouver, Canada’s Road to Recovery (R2R) initiative. This study explores healthcare staff experiences working within the new unit to identify opportunities to improve clinical service delivery and working conditions.

**Methods:**

This study employed a mixed method design. Between May and July 2024, R2R healthcare staff were invited to complete a cross-sectional survey to characterize their experiences working in a new hospital-based withdrawal management unit. A purposive sample of healthcare staff were selected to complete a one-time semi-structured qualitative interview between July and August 2024. Descriptive statistics were used to summarize survey data, while conventional content analysis was used to analyze qualitative data.

**Results:**

Thirty-three healthcare staff completed the study’s cross-sectional survey, including clinicians (e.g., nurses, physicians, pharmacists) and other staff such as social workers, peer support specialists, and Indigenous Wellness Liaisons (IWLs). Overall, 97% (*n* = 32) reported feeling satisfied with their role at R2R. Most respondents felt the model of care was well-rounded (82%, *n* = 27), trauma-informed (82%, *n* = 27), culturally and spiritually sensitive (85%, *n* = 28), and safe (94%, *n* = 31). Qualitative interviews (*n* = 8) revealed a consistent sense of pride in delivering ‘recovery-oriented’ care. Participants also felt R2R care provision was individualized, high-quality, and promoted meaningful engagement with patients through the cultivation of a safe environment. Job satisfaction was linked with feeling autonomous in providing patients access to a comprehensive spectrum of substance use services. Integration of traditional healing practices, as well as the incorporation of unique provider roles including peer support specialists and IWLs were identified as unique factors contributing to positive work experiences. Areas identified to be challenges included overly rigid approaches to patient care following relapses to substance use, as well as ongoing shortages of experienced, culturally safe healthcare staff, contributing to capacity constraints and burnout in the midst of the ongoing toxic drug crisis.

**Conclusions:**

Overall, healthcare staff reported job satisfaction working in a new hospital-based withdrawal management unit. Furthermore, participants described the R2R model of care as comprehensive, trauma-informed, and culturally safe.

**Supplementary Information:**

The online version contains supplementary material available at https://doi.org/10.1186/s13722-026-00705-y.

## Background

Healthcare staff in withdrawal management settings face growing challenges delivering effective care due to the increasingly toxic and unpredictable unregulated drug supply. Since 2016, over 400,000 people in North America have died from drug-related overdoses [1–5], largely driven by potent-substances such as fentanyl, in addition to other toxic adulterants including unregulated benzodiazepines and xylazine [3, 6–8]. Collectively, these substances intensify sedation, cause atypical withdrawal, and introduce complications such as seizures, amnesia, and skin lesions—rendering traditional withdrawal approaches increasingly ineffective [9–16]. In the absence of effective management strategies for addressing withdrawal in the fentanyl era, healthcare providers are facing unprecedented challenges. In community-based settings—where most withdrawal management occurs—staff often lack access to intravenous medications and the higher-level medical monitoring required for managing increasingly complex withdrawal syndromes stemming from growing toxic drug supply, including those involving seizures [17]. This creates a fragmented system of care, where patients are frequently escalated to hospital settings, disrupting continuity of care and adding strain to both patients and providers. As a result, healthcare workers are left to navigate care with limited tools, leading to mounting frustration and moral distress as they watch patients suffer —further exacerbating burnout and instability in the addiction care workforce.

In response to these challenges, an innovative addiction treatment model known as the Road to Recovery (R2R) Initiative was implemented in Vancouver, British Columbia, Canada [18–21]. The R2R model seeks to: (1) increase capacity to provide on-demand addiction care by trained health professionals; and (2) improve operational efficiency and coordination of existing clinical services to ensure patients can be seamlessly supported at every phase along their recovery journey [18, 19]. The R2R model provides an integrated, continuum-based system, linking on-demand acute stabilization with long-term recovery supports. Central to R2R’s clinical approach is a recovery-oriented model of care, which prioritizes patient autonomy, individualized goal-setting, and a non-linear understanding of recovery. The model is supported by a multidisciplinary team including physicians, nurses, pharmacists, social workers, peer support specialists, and Indigenous wellness liaisons. (IWLs), reflecting a commitment to incorporating diverse perspectives and promoting trauma-informed, culturally safe care. Cultural safety was embedded as a foundational principle in the design and implementation of R2R, including partnership with the health authority’s Indigenous Wellness and Reconciliation (IWRs) team from the earliest stages of program development. The model integrates novel roles intended to enhance cultural safety within substance use care, including Indigenous clinical providers such as IWLs and Indigenous Peer Support Specialists. These providers are integrated directly into the care team to support culturally grounded, relationship-based care, to promote comfort and trust among Indigenous patients. This includes facilitating access to traditional healing and cultural practices such as smudging ceremonies and talking circles, as well as incorporating culturally meaningful elements into the care environment, including traditional foods, natural elements within the unit space, and provision of culturally significant keepsakes such as smudged blankets.

As part of the R2R model, a hospital-based withdrawal management unit opened at St. Paul’s Hospital (SPH) in 2023. The R2R unit provides high-acuity inpatient withdrawal management for individuals with severe substance use disorders who are at greatest risk for substance-related complications, including withdrawal seizures and other complex medical needs. The increasing toxicity of the unregulated drug supply has led to more severe and medically complex withdrawal syndromes, creating a growing need for higher-acuity withdrawal management services embedded within acute care settings. Unlike conventional withdrawal management programs, a hospital-based setting allows for continuous medical monitoring, rapid access to acute and specialty care, administration of intravenous medications (i.e., IV hydromorphone, dimenhydrinate) and those requiring higher level monitoring (i.e., phenobarbital), and seamless transitions to ongoing treatment and recovery services following acute stabilization. Common pharmacologic interventions used include benzodiazepines for alcohol withdrawal management, short-acting opioids and clonidine for opioid withdrawal symptoms, and initiation or continuation of relapse-prevention therapies such as medications for opioid use disorder (MOUD).

Caring for people experiencing withdrawal presents a unique set of challenges for healthcare staff. Working in a high-stress environment with patients who have complex medical and psychosocial needs, responding to distressing events, and doing so with limited resources and (for some) marginal compensation can take a profound emotional toll—contributing to elevated levels of stress, moral distress, and burnout [22–24]. Leaves of absence and high staff turnover can further exacerbate compassion fatigue and burnout – resulting in significant shortages in the healthcare workforce [25–29]. Research examining job satisfaction, resilience, and compassion fatigue among healthcare staff in acute substance use settings is scarce to date [22]. Current research on healthcare work experiences predominantly focuses on the experiences of counsellors, harm reduction workers, and physicians in outpatient clinics or other community-based settings [22–24, 30, 31]. Few studies have formally characterized job satisfaction among substance use healthcare workers, nor gathered formal feedback on identified areas for improvement of substance use services in acute-care settings.

Recognizing the critical role that healthcare staff play in shaping patient experiences within substance use services, it is important to examine gaps in knowledge and practice. This study aims to understand the experiences and satisfaction of those working on the new hospital-based R2R withdrawal management unit and to identify areas for improvement in care delivery. As jurisdictions across North America and internationally seek to expand hospital-based withdrawal management capacity, understanding the staff experiences within these emerging models is essential for informing sustainable clinical service delivery.

## Methods

### Study summary

This study employed an explanatory sequential mixed method study design, in which the research team first collected quantitative survey data and then conducted qualitative interviews to deepen the understanding of survey findings. A transformative framework guided the study design, prioritizing the perspectives of staff serving marginalized populations and orienting the research towards identifying actionable opportunities to advance equity in substance use care delivery. The research team invited R2R operational and healthcare staff via email to participate in a cross-sectional survey about their individual experience providing substance use care as part of R2R’s new withdrawal management unit [32]. Data collection occurred between May and July 2024. In total, approximately 50 individuals were invited to partake in the healthcare staff survey in May of 2024, with one reminder e-mail invitation sent in July.

Following quantitative survey data collection, a purposive sample of healthcare staff were invited to participate in a semi-structured qualitative interview to explore their experiences in the field of addiction medicine, their perceptions of the R2R model of care and its implementation, and opportunities for how clinical services could be improved. Development of the qualitative interview guide was informed by preliminary findings from the cross-sectional survey and feedback from healthcare staff who completed the survey. Qualitative interviews occurred between July and August 2024.

The University of British Columbia (UBC)-Providence Health Care Research Ethics Board waived the need for a formal ethics application, as this project was deemed to be a quality improvement initiative. While Research Ethics Board approval was waived for this study; participation was entirely voluntary, and completion of the study materials was considered implied consent. Written informed consent was therefore not obtained as part of the study process.

### Study setting and participants

#### Clinical setting and model of care

The study takes place in the R2R’s newly opened withdrawal management unit in SPH, a tertiary care centre in downtown Vancouver, BC, Canada. Admission to the unit is voluntary however once admitted, patients are not permitted to freely move on and off the unit in an effort to minimize risk for destabilization and relapse. Within the R2R context, patients admitted for withdrawal management receive medically supervised treatment of acute withdrawal symptoms, which is further complimented by individualized care planning to facilitate transition to recovery-oriented after care programs aligning with patient’s self-stated goals, which can include community-based treatment (i.e., substance use care provided in outpatient settings such as addiction clinics or primary care settings, mutual peer-support programs), and/or more intensive recovery services including bed-based treatment. In the Canadian context, bed-based substance use treatment services typically refer to 30 to 90-day residential programs focused on psychosocial and recovery-oriented interventions (i.e., peer led group-based treatment), and the term ‘residential treatment’ has increasingly been avoided due to its association with the residential school system and the harms experienced by Indigenous communities.

Substance use is not permitted on the R2R unit. In the event this occurs, an individualized discussion is had to review recovery goals and potentially re-evaluate the care plan. Individuals referred for care at this site predominantly include: those at the highest risk for severe, complicated withdrawal (e.g., seizures, death) and/or have complex or sub-optimally managed comorbidities (e.g., end-stage organ failure, poorly controlled psychiatric diagnoses etc.) and may benefit from access to hospital-based consultative services (e.g., cardiac, renal, neurology), as well as identified priority populations (e.g., Indigenous, pregnant, and youth/young adults).

#### Participants

Eligible study participants included members of the interdisciplinary R2R healthcare team including: physicians, nurses, social workers, pharmacists, peer support workers, Indigenous Wellness Liaisons (IWL), care aides, unit clerks, and relational security staff. While the majority of healthcare staff invited to complete the survey predominantly included individuals providing direct care to patients on the withdrawal management unit, operational staff and leadership (e.g., clinical nurse managers, clinical educators, program managers) were also invited to participate.

#### Recruitment

Cross-sectional Survey invitations were distributed via email to R2R healthcare and operational staff in May 2024. Reminder emails were sent in July 2024 to maximize response rates. With support from PHC’s Interdisciplinary Substance Use program’s Medical and operational Directors, a purposive sample of healthcare staff was identified for qualitative interviews to ensure representation across occupational roles and varying levels of experience on the unit. After a potential participant list was developed, a copy of the project summary was sent to eligible R2R staff inviting them to participate in a semi-structured qualitative interview. Participants did not receive compensation for survey completion or interview participation.

### Data collection and measures

#### Road to recovery healthcare staff experience survey

A literature review was undertaken to identify previously validated questions and questionnaires used for assessing staff experiences in healthcare settings - where possible questions related to the provision of care in substance use were integrated. The survey was separated into distinct sections to capture a broad range of clinical work experiences. Domains evaluated in this survey included: workflow efficiency, clinical documentation, team dynamics, program views and perceived efficacy of the R2R model of care, and job satisfaction. Tailored questions were limited to the Workflow Efficiency domain; all other domains were identical across versions.

***Workflow Efficiency***: Questions explored staff experiences with new unit workflows (e.g., admissions, medication administration), focusing on daily R2R tasks such as patient intake, transitions and aftercare planning, communication during rounds, and follow-up. Surveys were tailored to occupational roles based on input from the R2R operational team and frontline staff, leading to different versions of the survey based on occupational role.

***Clinical Documentation***: Questions captured healthcare staff experiences with—including implementation training and administration of— a newly implemented substance use assessment and documentation tool integrated within the electronic medical record. The Brief Assessment at Intake and Discharge (BrAID) form is a standardized clinical documentation tool that is completed during intake by members of the R2R’s interdisciplinary care team and captures: sociodemographic, clinical, and substance use information. Used by the R2R interdisciplinary team, the BrAID form supports standardized and consistent data collection at first contact and serves as the foundation for ongoing monitoring. The BrAID is not a validated instrument.

***Team Dynamics***: Team dynamics were understood through questions assessing staff perceptions of teamwork, support, and communication within the organization. Questions explored how connected staff feel towards shared goals, whether they receive help from coworkers, and if they know how to access needed information. Additionally, the questions gauge openness to team building and whether staff feel their feedback is acknowledged and acted upon.

***R2R Program Views and Perceived Efficacy of the Model of Care***: Questions were informed by the adopted model for patient-centered substance use treatment— specifically items from the provider version of the Recovery Self-Assessment (RSA-R) were utilized [33]. Questions were intended to capture healthcare staff perceptions on the organizational mission and staff motivation working within the R2R model, role of the staff in creating a welcoming and comfortable environment, promoting hope and high expectations among patients in recovery, whether staff respect and support participants’ choices in their treatment, whether the program is perceived as having been designed around patient needs and preferences, and finally, whether staff are committed to maintaining a culturally safe environment for patients and their families.

***Job Satisfaction***: Questions were adapted from a validated measurement tool developed by Ahmad et al. (2020) for healthcare workers, and modified for experiences in substance use and withdrawal management [34].

The study’s survey featured Likert scale questions, multiple-choice questions, and open-ended questions, organized into sections for easier analyses and completion [35]. Most survey items used a 4-point Likert scale, consistent with the response formats of the validated instruments from which the questions were adapted. The 4-point scale included anchored responses ranging from low to high agreement/frequency, depending on the item. The clinical documentation section was the only exception and used a 5-point Likert scale to better capture variation in documentation-related experiences. The survey was pilot tested with four healthcare staff members to identify ambiguities, assess clarity and relevance of items, and evaluate overall survey flow. Based on feedback from pilot participants, several multiple-choice response options were expanded to better reflect the range of clinical and workplace experiences reported by staff (i.e., tasks across roles), and additional questions were added to capture experiences that were not adequately represented in the initial draft survey. Surveys were accessible through a link provided through email, administered using REDCap, a secure web-based platform for research data capture, ensuring data security and ease of access [36].

#### Road to recovery healthcare staff qualitative interviews

A purposive sample of R2R healthcare staff were then invited to complete one qualitative interview, which included physicians (attendings and residents), nurses, social workers, peer support specialists, IWLs and members of the clinical operations team. Interviews took place between July and August 2024. Participants were invited to describe their experiences working with R2R and provide feedback on how these services could be improved. Interviews were conducted one-on-one, lasted approximately 30 min on average and took place in-person at St. Paul’s Hospital. Interviews were conducted by a summer intern involved in the R2R program evaluation team. The interviewer received training in qualitative interviewing and data collection from the qualitative team lead (J. Bowles), as well as ongoing supervision from the study co-PI (B. Dennis). The interviewer was affiliated with the broader program evaluation team but was not involved in direct clinical care or participant supervision. Interviews were completed using a semi-structured interview guide (See supplementary appendix), which was designed to explore both a priori (anticipated survey content) and unexpected content that emerged during the interviews, and probe accordingly. Data consisted of audio-recorded interviews transcribed verbatim by members of the research team. Transcripts and audio recordings were password-protected and stored on encrypted storage devices. Hard copies of study documents (i.e., notes from interviews, demographics survey from interviews) were stored in locked filing cabinets within the study office.

### Analysis

#### Road to recovery healthcare staff experience survey

Descriptive statistics were used to summarize the baseline demographic and healthcare staff survey data. Medians and interquartile ranges (IQR) were calculated and reported for continuous variables. Frequencies and percentages were used to report categorical variables. Given negligible missing data, no imputations methods were used. All analyses were completed in Microsoft Excel [37].

Open-text survey responses were reviewed by members of the R2R evaluation team to identify common ideas, experiences, and areas of feedback relevant to the evaluation objectives. Responses were analyzed using a descriptive content analysis approach, with recurring concepts grouped into broader categories and incorporated into the overall interpretation of findings.

#### Road to recovery healthcare staff qualitative interviews

Two coders reviewed manuscripts and contributed to content analysis. One coder conducted interviews. Discrepancies were handled through mediated discussion with the qualitative study lead (J Bowles).

A hybrid approach to content analysis was used, combining deductive and inductive analytic strategies. Broad areas of inquiry were established a priori based on the study’s primary objectives, including healthcare staff experiences, perceptions of the R2R model of care, evaluation of interventions perceived to be working well for patients, and identification of what is working well for priority populations (including Indigenous and youth populations) accessing services. Within these domains, specific codes and themes were derived inductively from the data through open coding, followed by a round of focused coding to refine emerging themes. Key concepts and themes, along with illustrative examples, were identified, compared, and contrasted across the entire dataset.

Regular meetings were undertaken to review new data, which was subsequently compared to emerging analytic concepts for refinement using a constant comparative approach [38].

## Results

### Healthcare staff experience survey results

A total of 33 healthcare staff completed the study’s cross-sectional survey between May and July of 2024 (Table 1), of which 70% (*n* = 23) identified as cis-women, 46% (*n* = 15) identified as White, the median age was 33 years (IQR 29–40) and 76% (*n* = 25) reported previous work experience in the substance use field.

**Table 1:** Demographic characteristics of healthcare staff working as part of the R2R’s hospital-based withdrawal management unit who completed the study’s cross-sectional survey completed between May and July of 2024 (*n* = 33)

Section A: Demographics	
Professional role at R2R	**n (%)**
Nurse	15 (45)
Doctor	7 (21)
Unit Clerk	3 (9)
Care Aide	3 (9)
Peer Support Worker	2 (6)
Pharmacist	1 (3)
Social Worker	1 (3)
Security	1 (3)
**Length of experience in R2R role (months)**	***n*** **(%)**
7–12 months	16 (49)
3–6 months	12 (36)
< 3 months	5 (15)
**Previous substance use care experience**	***n*** **(%)**
Yes	25 (76)
No	8 (24)
**Length of experience in substance use care (if answered ‘Yes’ above) (*n*** **= 25)**	***n*** **(%)**
< 1 year	3 (9)
1–5 years	15 (46)
6–10 years	4 (12)
> 10 years	3 (9)
Not applicable	8 (24)
**Gender identity**	***n*** **(%)**
Man	8 (24)
Woman	23 (70)
Other responses: non-binary and prefer not to say	2 (6)
**Ethnicity**	***n*** **(%)**
White	15 (46)
Other Asian	8 (24)
South Asian	4 (12)
Chinese	3 (9)
Other categories: Black African or Caribbean, Middle Eastern, Polynesian	5 (15)

Table Caption: Percentages may not sum to 100 due to rounding and/or participants selecting all options that applied. Demographic characteristics that could be identifying for respondents have been combined and include specific gender and ethnicity subcategories

#### Workflow efficiency

**Fig. 1:**
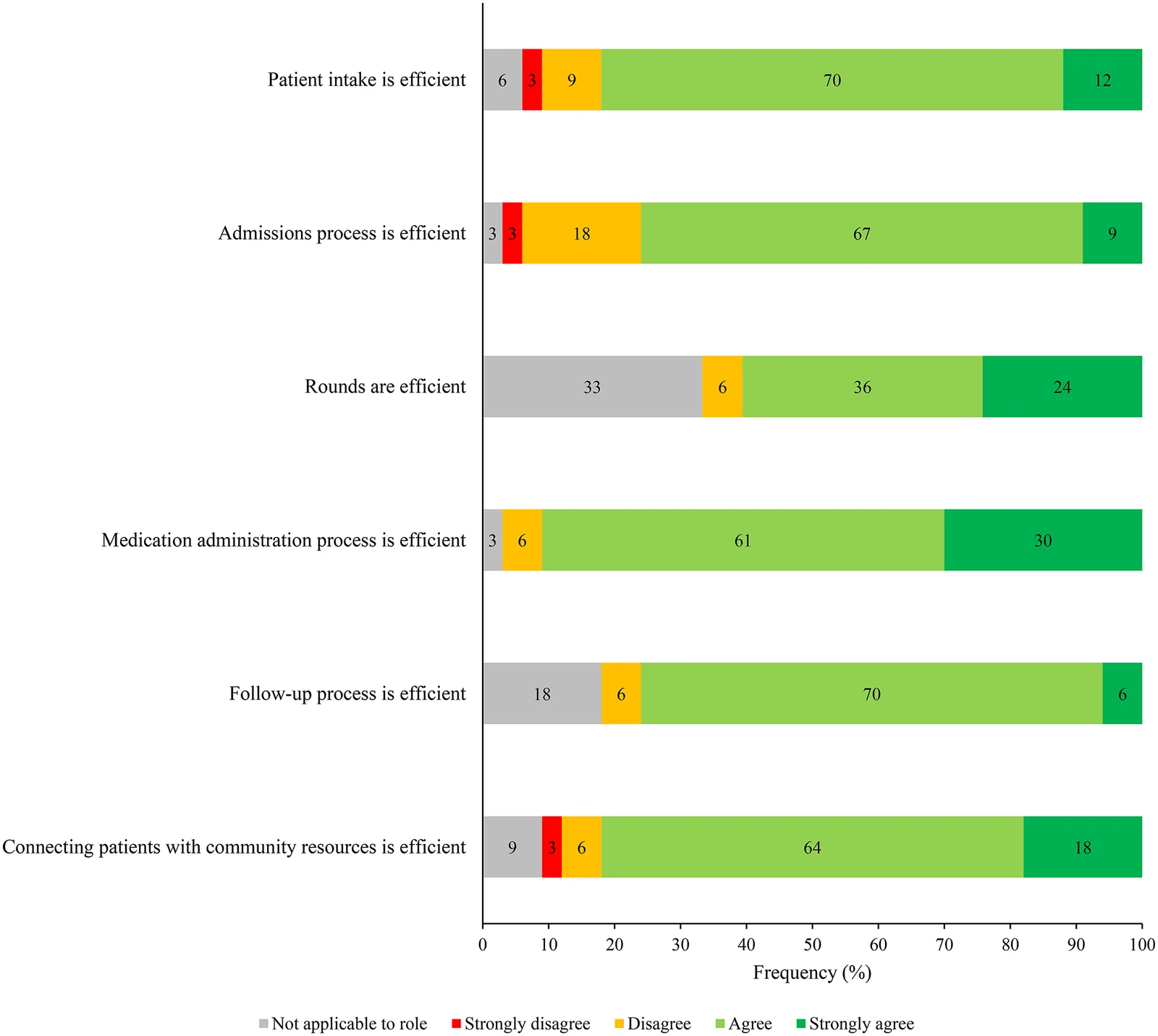
Healthcare staff from SPH’s R2R withdrawal management unit’s perspectives of Workflow efficiency from cross-sectional survey, May-July, 2024 (*n* = 33). Figure Caption: Percentages may not sum to 100 due to rounding

The majority of healthcare staff agreed or strongly agreed that workflow tasks across the patient journey, from intake processes (82%, *n =* 27), rounds (61%, *n =* 20), medication administration (91%, *n =* 30), follow-ups (76%, *n =* 25), and discharge planning (82%, *n =* 27) were efficient (Fig. 1). Among the most prominently surveyed group which was nurses (*n* = 15), the workflow tasks perceived to be most efficient included: vital assessments (80%, *n* = 12), shift report (67%, *n* = 10), hourly patient assessments (53%, *n* = 8) and hourly pain medication distribution (47%, *n =* 7) (Table 2).

**Table 2:** Perceived facilitators and barriers impacting workflow efficiency among R2R nurses at SPH from cross-sectional survey, May-July, 2024 (*n* = 15)

Workflow Efficiency	
**Perceived facilitators to intake efficiency** ^*^	**n (%)**
Vitals	12 (80)
Getting patient set up in the unit (showering, sorting belongings, etc.)	5 (33)
Full assessment	5 (33)
Filling out the BrAID^†^ form	2 (13)
Other	2 (13)
**Perceived barriers to intake efficiency** ^*^	***n*** **(%)**
Patients experiencing distress on the unit can activate patients being admitted and undergoing intake	12 (80)
Filling out the BrAID^†^ form	8 (53)
Waiting for patients to finish showering	8 (53)
Intake patients coming early	8 (53)
Previous patient has not been discharged yet	5 (33)
Other	5 (33)
Full assessment	4 (27)
Vitals	1 (7)
**Perceived facilitators in overall nursing workflow** ^**‡**^	***n*** **(%)**
Shift report	10 (66)
Hourly assessments	8 (53)
Hourly pain medication distribution	7 (47)
Check in	5 (33)
8am medication distribution	5 (33)
Smoke break	4 (27)
Breakfast	4 (27)
**Perceived barriers in overall nursing workflow** ^**||**^	***n*** **(%)**
Other	8 (53)
Hourly assessments	4 (27)
8am medication distribution	3 (20)
Smoke break	3 (20)
Hourly pain medication distribution	2 (13)
Shift report	1 (7)
Check in	1 (7)
Breakfast	1 (7)

Table Caption: Survey questions presented in Table 2 completed only by nursing staff (*n*=15). Percentages reflect a denominator of 15 and may not sum to 100 due to rounding and/or participants selecting all options that applied. Imputations for incomplete responses presumed respondents did not check any items for these questions. *Missing data: *n*=1; †Brief Assessment at Intake and Discharge; **‡**Missing data: *n*=2; **||**Missing data: *n*=3

Among all respondents, admission processes were found to be the least efficient part of the R2R daily workflow (i.e., 21% of respondents either disagreed [18%, *n* = 6] or strongly disagreed [3%, *n* = 1] that admissions processes were efficient) (Fig. 1). Among nurse respondents, the most common barrier to patient intake efficiency during the admission process included patient-patient interactions (e.g., existing R2R patients may distress and/or trigger new patients being admitted to the unit, 80%, *n* = 12).

#### Clinical documentation

**Fig. 2:**
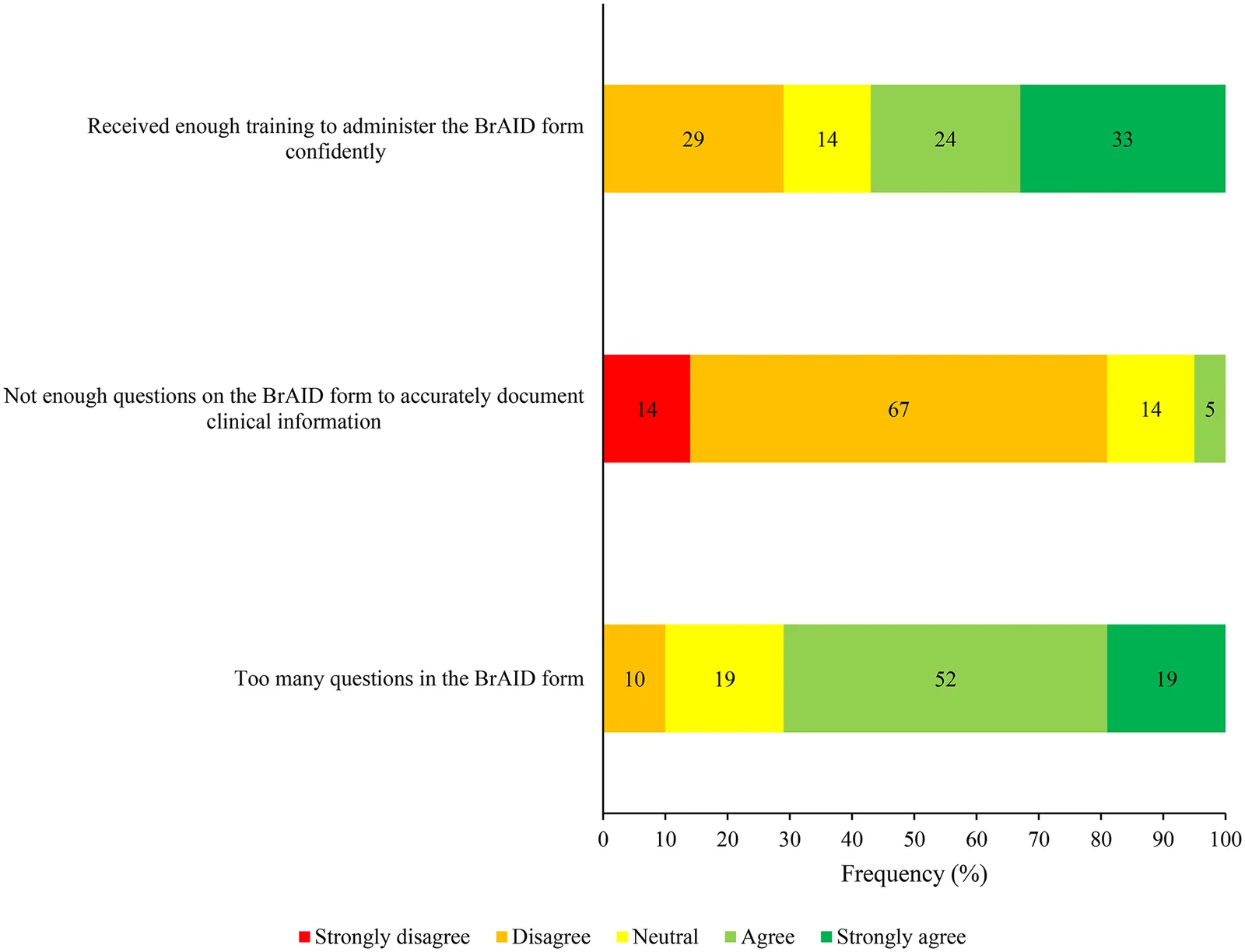
Healthcare staff’s clinical documentation experiences administering the BrAID form on SPH’s R2R withdrawal management unit from cross-sectional survey, May-July 2024 (*n* = 21). Percentages may not sum to 100 due to rounding

Among staff involved in completion of the BrAID form (*n* = 21), 71% (*n =* 15) reported this substance use assessment tool collected useful clinical information, whereas 43% (*n* = 9) felt additional support (and/or incentives) would aid in consistent completion of the form and better integration within the existing clinical workflow. More than half of respondents (57%, *n* = 12) either agreed or strongly agreed that they received enough training to complete the BrAID form, with 71% (*n* = 15) of participants felt there were too many questions included (Fig. 2).

#### The R2R model of care: perceived program views and efficacy

**Fig. 3:**
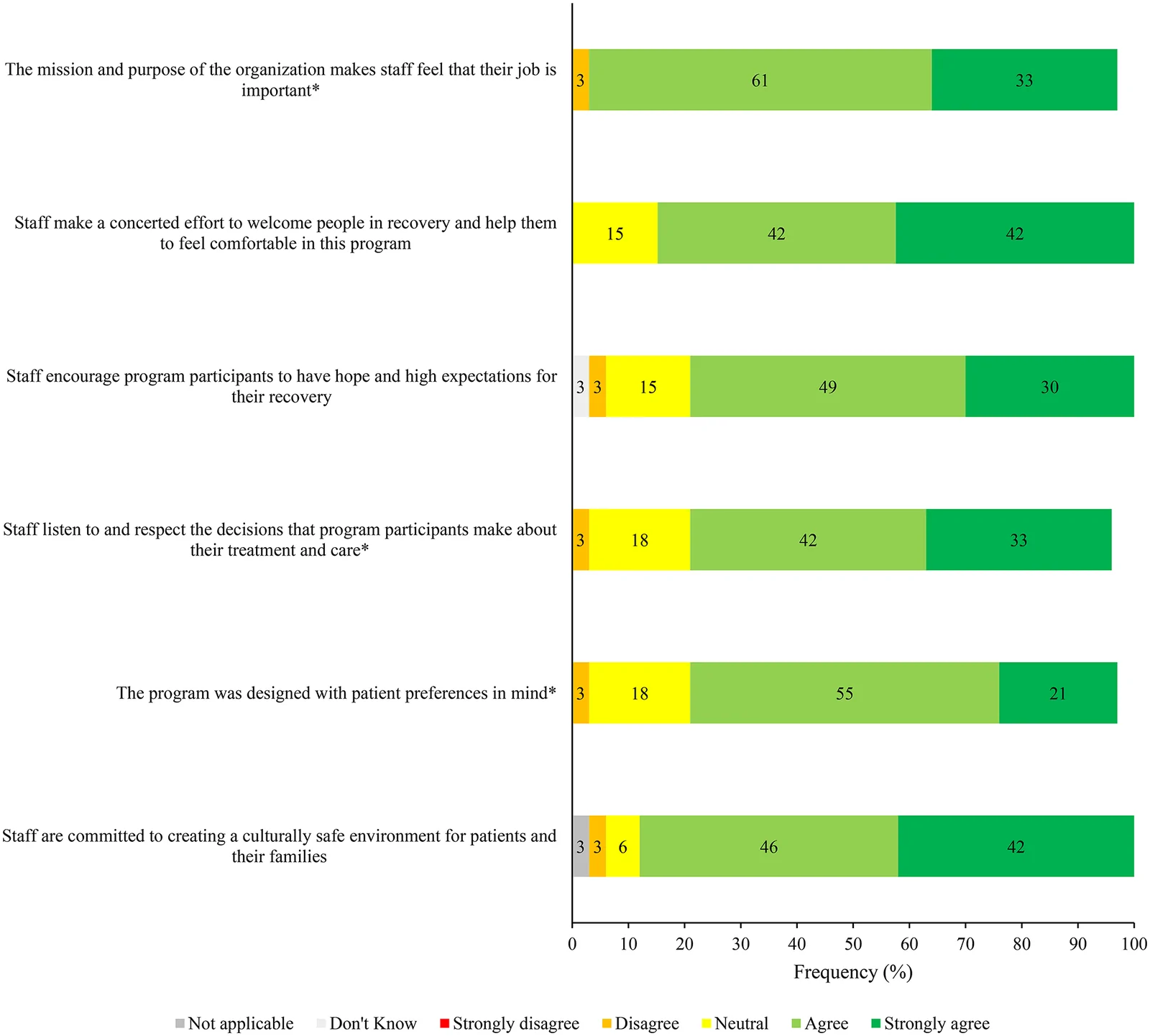
Healthcare staff from SPH’s R2R withdrawal management unit’s perspectives on the program’s views and its efficacy from cross-sectional survey, May-July 2024 (*n* = 33). Figure Caption: Percentages reflect a denominator of 33 outside of identified cases and may not sum to 100 due to rounding. Imputations for incomplete responses presumed respondents did not check any items for these questions. *Missing data: *n*=1

Participants highlighted that they felt there were numerous reasons why R2R may be different than other substance use services (Table 3; Fig. 3). Three quarters of respondents (76%, *n* = 25) felt the R2R model of care was designed with patient preferences in mind and respected patient values and decisions regarding their treatment. Approximately 79% (*n* = 26) of respondents felt the R2R model of care encouraged hope. Further, 88% (*n* = 29) felt R2R staff were intentional about creating a culturally safe environment (especially for Indigenous patients) and made a concerted effort to welcome patients onto the unit to foster comfortability (85%, *n* = 28). When compared to what would be available through traditional community-based withdrawal management sites, over three-quarters of respondents (76%, *n* = 25) felt R2R improved access to a wider number of substance use and bed-based treatment services (i.e., connection to specialized tertiary care substance use treatment sites or treatment centres requiring specialist assessment), 70% (*n* = 23) responded R2R facilitates better connections to community and subspecialty care, 82% (*n* = 27) responded that care is more well-rounded, and 94% (*n* = 31) found the model of care to be safe. Other unique aspects identified included: more privacy for patients, adoption of more patient-centered approaches to care (i.e., clinical decision making, recovery planning), more opportunities for psychosocial treatment, recovery housing and transitional care beds provided, faster access to detox services, and access to employment and vocational training opportunities as shown in Table 3.

**Table 3:** Healthcare staff from SPH’s R2R withdrawal management unit’s perspectives on the program’s views and its efficacy from cross-sectional survey, May-July 2024 (*n* = 33)

Program Views	
Need for better approaches to the design and evaluation of the R2R services*	***n*** **(%)**
Yes	8 (24)
No	22 (67)
**All appropriate partners were involved in the decision-making process when developing and assessing the R2R?** ^†^	***n*** **(%)**
Yes	24 (73)
No	5 (15)
**Stakeholders that were felt to be not adequately involved (if answered ‘No’ above,n = 4).** ^**‡**^	***n*** **(%)**
Pregnant	3 (75)
Youth	3 (75)
Indigenous	1 (25)
Other	1 (25)
**Efficacy**	
**Descriptors of the R2R model of care**	***n*** **(%)**
Safe	31 (94)
Patient-centered	30 (91)
Culturally and spiritually sensitive	28 (85)
Trauma-informed	27 (82)
None of the above	1 (3)
**Unique differences of R2R compared to other experiences in substance use care**	***n*** **(%)**
Transitional care beds provided	28 (85)
Care is more well-rounded	27 (82)
Access to a wider number of treatments	25 (76)
Better connections to community and subspecialty care	23 (70)
Faster access to care	23 (70)
More patient-centered	21 (64)
Recovery housing provided	18 (55)
More privacy for patients	16 (49)
More opportunities for psychosocial treatment	11 (33)
Certain activities are more available	4 (12)
Access to employment/vocational services	4 (12)

Table Caption: Percentages reflect a denominator of 33 outside of identified cases and may not sum to 100 due to rounding and/or participants selecting all options that applied. Imputations for incomplete responses presumed respondents did not check any items for these questions. *Missing data: *n*=3; †Missing data: *n*=4; ‡Percentage adjusted using denominator *n*=4

Three-quarters of respondents (75%, *n* = 24) either disagreed or strongly disagreed that negative attitudes and/or stigma towards substance use are prevalent on the unit (amongst both staff and/or patients).

#### Team dynamics

**Fig. 4:**
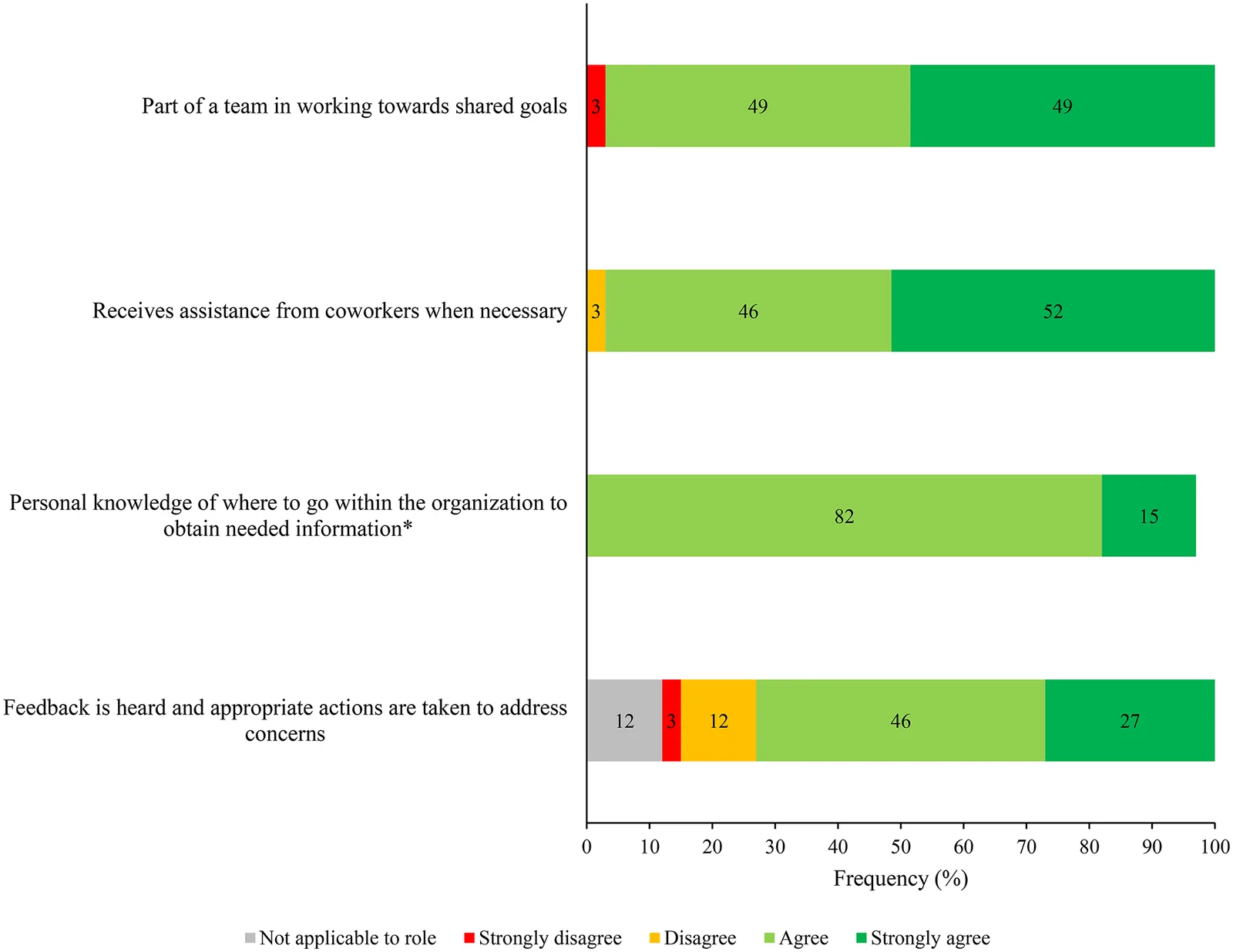
Healthcare staff’s perceptions of team dynamics on the R2R withdrawal management unit at SPH from cross-sectional survey, May-July 2024 (*n* = 33). Figure Caption: Percentages reflect a denominator of 33 outside of identified cases and may not sum to 100 due to rounding. Imputations for incomplete responses presumed respondents did not check any items for these questions. *Missing data: *n*=1

Overall, a strong sense of teamwork was reported among respondents (Fig. 4). More specifically, 97% (*n* = 32) agreed or strongly agreed they felt they were part of a team that was working towards shared goals, received assistance from coworkers when needed, and had a strong understanding of the organization’s structure. Almost three-quarters of respondents (73%, *n* = 24) either agreed or strongly agreed that they felt their feedback and concerns were heard and appropriate actions were taken to address those concerns. Beyond this, a high degree of interest was expressed in team-building exercises with 85% (*n* = 28) of respondents expressing interest in either developing or implementing exercises to further improve and strengthen dynamics within the R2R team.

#### Job satisfaction

**Fig. 5:**
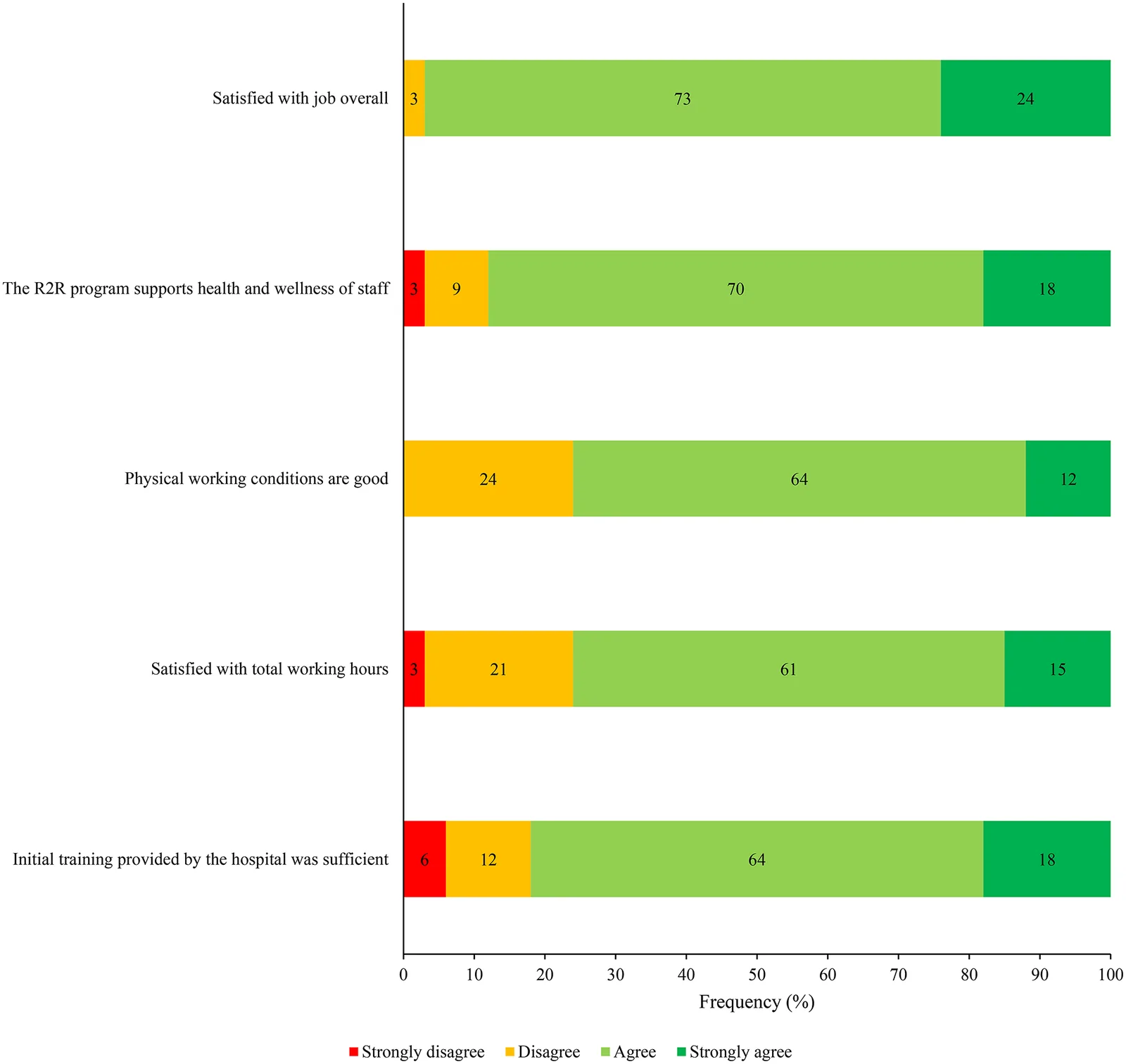
Job satisfaction among healthcare staff at the R2R withdrawal management unit at SPH from cross-sectional survey, May-July 2024 (*n* = 33)

A high degree of overall job satisfaction was reported (97%, *n* = 32) by R2R respondents. Furthermore, 88% (*n* = 29) felt R2R leadership supported the health and wellness of their staff and that initial training was sufficient (82%, *n* = 27). Staff identified protected break times (54%, *n* = 18), team events (48%, *n* = 16), and culturally safe environment training (48%, n-16) as key strategies to further support staff wellbeing (Table 4). Figure 5 presents a summary of job satisfaction among different healthcare providers.

**Table 4:** Identified supports that aid with job responsibilities and personal wellness among healthcare staff at the R2R withdrawal management unit at SPH from cross-sectional survey, May-July 2024 (*n* = 33)

Job Satisfaction	
**Additional supports that would help with job responsibilities** ^*^	**n (%)**
Mentoring/additional training related to ensuring a culturally safe environment for Indigenous patients	16 (48)
Regular huddles for refreshing standard practice	16 (48)
Additional hiring for addiction medicine educator hospital-wide	15 (45)
Monthly meetings with RNs and managerial staff	14 (42)
Hiring more staff in this role	14 (42)
Adequate office space	14 (42)
More check-ins with managerial staff	10 (30)
Formal training specific to the unit	10 (30)
Clerical support	10 (30)
Other	2 (6)
**Additional supports that would help with personal wellness** ^†^	***n*** **(%)**
Protected break times	18 (54)
Team events to increase team spirit	16 (48)
Additional hiring for this role	13 (39)
Additional opportunities for socializing during or after work	10 (30)
Other	2 (6)

Table Caption: Percentages reflect a denominator of 33 outside of identified cases and may not sum to 100 due to rounding and/or participants selecting all options that applied. Imputations for incomplete responses presumed respondents did not check any items for these questions. *Missing data: *n*=2; **†**Missing data: *n*=7

### Healthcare provider qualitative interview findings

#### Participant demographics

Eight participants completed a qualitative interview. Among these participants were nurses, those in leadership/administrative roles, peer support workers, social workers, Indigenous wellness liaisons, and physicians. Occupational roles not represented included administrative unit clerks, janitorial, and security staff. The median age of participants was 37 years (IQR 35–45), almost two-thirds (63%, *n* = 5) identified as being White, and 50% (*n =* 4) identified as women. On average, participants had worked on the R2R withdrawal management unit for 9.4 months, encompassing close to the entire duration of the units’ operational time (opened 10 months at the time of interviews). The majority of staff (88%, *n =* 7) reported previous experience working in the field of substance use with 63% (*n =* 5) and 38% (*n =* 3) reporting 1–5 years and 6–10 years of previous work experience, respectively.

#### Qualitative analysis

Findings were organized into four categories: (1) recovery-oriented care, (2) the R2R model of care, (3) facilitators and barriers to providing effective substance use care, and (4) a pathway forward to better managing the medical and psychosocial needs of those affected by the ongoing substance use crisis.

In general, participants expressed a strong sense of pride in their roles and the model *being patient-centered*,* it gives me joy and a certain level of purpose to help people find short- and long-term goals (and) obtain those goals (Respondent 1) and importantly furthered that* “*it is also so cyclical; so as rewarding as it can be it is … it can be disheartening when that falls through*, suggesting the impact witnessing unjust and, at times, uncontrollable barriers that disproportionately impact people who use drugs can have emotionally. Nonetheless, healthcare staff described a collective sense of pride in striving to deliver high-quality care that honours individuality and preserves dignity. R2R’s trauma-informed approach was described as promoting a safe and trusting environment, which facilitated positive experiences for staff and fostered hope for the future of substance use care, they agreed understanding the impact from the patients’ perspective remains crucial. Participants universally appreciated their ability to work in this field and serve and uplift patients during a vulnerable moment such as being in an acute substance use treatment space.

#### Recovery-oriented care

Even with the diversity in participants’ roles, all participants described “recovery-oriented” care as individualized, patient-centered, and ultimately defined by the patient. Central to these factors are the principles healthcare staff believed to be key in providing effective recovery-oriented care, which included elements such as compassion, empathy, honesty, and openness. Participants consistently used the phrase *meeting the patients where they are at*, which suggests that recovery-oriented care was understood not as exclusively abstinence or harm-reduction oriented paradigm per se, but as flexible and responsive to each patient’s unique goals and capacities, as highlighted by one participant:

*Recovery for someone is very different and often the first step is going to detox [for] some people*,* but I think it’s really meeting people where they’re at and having this conversation about what does that look like for them. I don’t think there’s one model for everyone. I think everyone comes with their own personal journeys and relapse is part of the recovery too” (Respondent 2)*

Another participant agreed, stating that patients define recovery on their own terms and emphasized the need to respect their substance use decisions and goals, even when they choose to prematurely self-discharge from R2R:

*It’s very individualised… every persons’ goals are different*,* and it takes a different path to get there. Even if a person comes and self-discharges*,* I still think that’s a win because … they got to come and learn some things (Respondent 4)*

Reframing self-initiated discharge by focusing on what patients gained during their time at R2R, rather than what they lost by leaving *prematurely*, might have aided in the morale of participants. Moreover, participants described a strong commitment to fostering an environment of understanding, acceptance, and non-judgment, with the aim of promoting relationship-centered, recovery-oriented care while also being realistic about outcomes given the chronic, remitting, and relapsing nature of the disease. A key to honoring recovery-oriented care that is patient-centered was redefining the metrics of what successful recovery means:

*I mean*,* I could easily say to you*,* like the biggest rewarding thing is when someone finishes the program and does well*,* that is going to throw us all through a loop if that is our measure of success*,* because addiction is like a relapsing process*,* and I used to feel like that was my measure of success when I first started treatment. And now I’ve like really come to this space for a while that the most rewarding thing is really just my interactions with patients. (Respondent 7)*

#### Road to recovery model of care

The R2R model of care was consistently described by respondents as a *wraparound care* characterized as highly resourced and accessible, with comprehensive supports to facilitate seamless transitions as across the complete substance use continuum of care. One participant summarized R2R as:

*Using trauma-informed care strength-based conversation*,* meeting them where they’re at*,* understanding substance use and mental health*,* where it’s coming from*,* and just seeing them as a human being (Respondent 3).*

Participants emphasized that the R2R model of care stands out due to its comprehensive approach, offering access to acute-level care, rapid physician and specialty-care assessments, and a multidisciplinary team, e.g., *the beauty of Road to Recovery is that we do have the capacity to do bloodwork*,* we do have the capacity to call specialists to come and help us if there’s a specific question that needs to be addressed (Respondent 5).* They highlighted that access to vital services such as treatments for comorbid conditions, performing procedures, and even providing reproductive care were key and not their experience in other settings:

*We are able to connect them in ways that we’ve never been able to before …. we just accept people for what they want to do*,* which is detox*,* but at the same time we can treat their hepatitis C*,* we can treat their cardiac valve disease*,* we can treat their pneumonia*,* we can optimize their diabetes*,* their blood pressure. We can diagnose them with medical conditions that they don’t know they have that could be harmful if they continue to live with them for a long period of time so it’s* [19] *very unique for that. (Respondent 5)*

Participants described that the wraparound services offered by the R2R model, supported by a large multi-disciplinary team where a diversity of perspectives, including individuals with lived or living experience and expertise of substance use, were involved in decision making and integrated into overall patient care: e.g., *to just have a full healthcare team behind the recovery process is something these other places that really can’t afford or don’t have that within their program kind of sets them* [19] *apart from everybody else. (Respondent 1).* This sentiment was supported by other participants:

*I think it was really great that they included a peer perspective*,* someone with lived experience*,* and I felt like my input was valued by the team and I noticed that just when I would make suggestions*,* there wasn’t any pushback or anything like that. (Respondent 4)*

Participants noted feeling they worked in an environment where it was not only safe to be open about their lived experience with drug use but was also culturally safe for Indigenous patients:

*As an Indigenous Wellness Liaison*,* we’re not seen differently because of our roles. We are a team. And that’s how I see it. And that’s what I feel. And that’s what we do*,* even with the addiction doctors. It’s just so amazing. (Respondent 8)*

#### Facilitators and barriers to providing effective substance use care

Recovery-oriented care and the R2R model in general were viewed as facilitators of effective care. Participants often viewed effective care as treating individuals with respect and seeing them as whole people rather than reducing them to their substance use. A strong facilitator of R2R was its ability to adapt and be flexible to individual needs:

*It’s like everybody is so different and their needs are so different. I think that building flexibility into the program and not so much focusing on what the ultimate rules are*,* but kind of making decisions with the whole team on a case-by-case basis (Respondent 4)*.

While participants unanimously agreed that care should be patient-centered, a tension existed with respect to the operation and logistics of a unit comprised of patients with diverse needs, goals, and desires. As such, some discussed the importance of clear and consistent practices to address this potential barrier and ensure equitable care and maintain trust among all patients. This tension was explained by a participant who stated:

*It has to be really black and white and then other people are like*,* oh*,* there’s*,* you know*,* people should have a little bit more discretion….It just creates that distrust and then that’s not trauma-informed for patients and that’s like because there’s all this conflict about what’s going to happen and nobody knows what’s going to happen. And it’s not safe for staff*,* or it doesn’t feel like it doesn’t*,* like*,* then work becomes a place where people are like*,* oh*,* it’s super unpredictable. I don’t feel comfortable here*,* or like*,* I don’t know what I’m going to do in this tough situation. I don’t know if my team is going to have my back. (Respondent 2)*

As noted by one participant who described discomfort with the hospital culture in which the R2R is situated when a patient decided to initiate their own discharge:

*A large portion of the [general hospital staff] are very unaccepting of people who struggle with their substance use*,* if you are still wanting to use substances this is not the place for you and while I can empathise*,* we put patients in very precarious situations overnight in terms of their safety (Respondent 1).*

The most notable barrier to providing effective substance use care included the enduring stigma around substance use, which can deter patients from seeking potentially life-saving care, affect their self-image and recovery or healing pursuits, as well as erode trust with services and other health treatments/professionals. Participants often conceptualized substance use and addiction using a medicalized model, as opposed to a deviancy, noting disparities in the perceptions between other often stigmatized conditions and substance use disorders:

*We actually need to address it as a medical condition. We don’t tell people*,* stop having diabetes. Stop having HIV*,* stop having Hep C. We treat it. So*,* we need to start treating people that are sick with medical treatment that we do know works. (Respondent 5)*

Participants reported feeling stigmatized due to working in the field of addiction, which might impact how they feel in their profession:

*Stigma and often the addiction medicine physician are stigmatized in some degrees the same way as the patients are because we work with that population so often*,* even with our colleagues in different subspecialty*,* we are not felt like we are a real* physician….I *just feel it’s part of the stigma and working with a stigmatized population*,* you are unfortunately sometimes in the scope of being stigmatized. (Respondent 5)*

Further barriers impeding the provision of effective care included the pronounced sense of burnout and emotional stress felt among substance use staff. Participants acknowledged the workforce shortage impacting substance use care and described consistent factors contributing to this shortage including burnout, perceived stigma, emotional stress from being direct witnesses of a public health emergency, limited leadership opportunities in certain professions, in addition to poor compensation:

*I mean*,* in the context of the toxic drug emergency*,* there’s been so much loss. We’ve lost so many people. I mean*,* it’s been going on for like eight years now. So I think*,* you know*,* maybe there’s like a sense of like futility. And also just a heartbreaker on that (Respondent 1* **)** [39, 40].

Finally, the inability to provide effective discharge planning and continuity of care due to persisting systemic barriers outside of the staff’s control left many feeling a sense of futility in efforts to provide care, further escalating rates of burnout:

*I think that it’s tough to see people struggle every day. And sometimes that can get a little overwhelming*,* especially if you feel like there’s resources that are not being offered. That can be incredibly frustrating if you know what will help a person*,* but for whatever reason you aren’t able to provide that*,* I think that can… so yeah*,* I think burnout is a huge thing in this field. (Respondent 7)*

#### Suggestions for improvement of R2R model of care

Increased capacity was identified as an important way to reduce waitlist times while also ensuring structurally vulnerable patients are guaranteed recovery-oriented housing and care through all stages of their recovery and healing pursuits:

*I mean*,* we need more treatment beds up to 100%. Like*,* without fail*,* that needs to be like a huge priority for like government*,* more treatment beds. Yeah. And then we need more harm reduction approaches*,* or harm reduction services*,* like housing services. And maybe more housing*,* like you’d like to not do this. Like you can’t even talk about addiction without talking about the need for housing*,* right? Like every part of our patients issues with navigating or like addiction and mental health is around lack of housing*,* and safe housing. (Respondent 7)*

Furthermore, staff recognize a need for increased capacity in terms of space and team members. There is a recognition of the interdisciplinary team being a core strength of the success of R2R, and as such, increasing the number of available team members to facilitate more individualized care plans:

*We would need more pharmacists. We would need way more social workers… I think we need 24-hour peer services and we need more than one peer at a time. Like peers are undervalued resources… I think we need more culturally sensitive areas (culturally safe spaces for Indigenous patients). It would be awesome if we have more*,* again*,* if we have a bigger space*,* but if we had more room where our Indigenous Liaison [IWL] can have this smudging session*,* then the different rituals that they want to have to help the Indigenous population just through the phase of ritual and reconnecting with their peer as well. (Respondent 5)*

More staff were also seen as a gateway to intervention in greater depth:

*I mean*,* personally*,* obviously*,* I really want another social worker to be hired in the like*,* in the our five*,* like*,* fifth floor unit. Because it would open up so many cool doors to do more groups more counselling*,* like more assessment*,* like proper assessment of pathways*,* more time to connect with someone more motivational interviewing conversations*,* deeper and more effective intervention. (Respondent 7)*

Additional staff were also viewed as having the ability to provide the unique services more frequently:

*I’d love to do the Monday to Friday but also with another team member*,* an IWL. It takes two because I could work on one end medically to understand and then if I had a team member that would work with the cultural stuff that they need. So because it’s all on me when I bring it in. (Respondent 8)*

Given the breadth of staff involved in R2R, some participants felt clarification of healthcare staff roles and exploration of new ways to more effectively integrate certain team members could lead to more successful outcomes. This was particularly evident for the peer role:

*I think the peer support can be more valued and more utilized in the care*,* especially here*,* I feel like doctors talks to patients individually*,* nurses talk to patients individually*,* and then I go in and talk to them. Everybody that talks to patients individually*,* but not with the peer worker (Respondent 3)*.

## Discussion

Our findings highlight the experiences of healthcare staff working within an innovative substance use treatment program in an area characterized by high rates of substance use harms. With the implementation of R2R’s new model of care, a timely opportunity emerged to conduct a mixed-methods evaluation to characterize the experiences of healthcare staff in the early stages of program implementation. The majority of survey participants described overall satisfaction with their role at R2R and expressed pride in providing high-quality care in congruence with R2R’s values of trauma-informed and person-centered care. Overall, the degree of healthcare staff job satisfaction was mirrored by high levels of team engagement, workflow efficiency, and positive beliefs in the quality of the care and benefit from direct involvement in each patient’s recovery journey. Similar to findings from the survey data, qualitative interviews revealed participants universally described R2R’s service delivery as high quality and as a result of a collective effort towards a recovery-oriented care model. Participants consistently described recovery-oriented care as flexible, individualized, and defined by patient goals rather than adherence to a single abstinence- or harm-reduction-oriented framework. This finding has important implications for hospital-based addiction medicine, where patients often present with highly complex medical and psychosocial needs. Rather than conceptualizing recovery as a linear process, participants emphasized the importance of building trust, maintaining therapeutic relationships, and supporting patients through fluctuating recovery trajectories that may include relapse or self-initiated discharge. These findings align with emerging recovery-oriented frameworks that emphasize patient autonomy, relationship-centered care, and individualized treatment planning within substance use services.

Additionally, the uniqueness of R2R’s comprehensive and culturally safe approach to addiction treatment was juxtaposed by participants’ previous work experiences in the substance use field. The inclusion of unique perspectives such as the Indigenous Wellness Liaisons and Peer Support Specialists was frequently highlighted as an important feature of R2R.

Conversely, healthcare staff also highlighted major challenges in providing high-quality care, which may ultimately impact the work environment and job satisfaction. As a response to the growing demands for substance use services amid the worsening public health emergency, R2R was created alongside other substantial resources and coordinated efforts in North America [14–21, 35, 36, 39]. However, as a consequence of the increasing need for these services, the strain on the health workforce has similarly increased, leading to higher staff turnover, compassion fatigue, and burnout [22, 23, 26–28]. Psychological stress, grief, and burnout were repeatedly identified as one of the primary challenges to providing effective care. Further, persisted workforce shortages, non-competitive wages, and perceived stigma around substance use amongst other healthcare staff have exacerbated this emotional toll on staff and, by extension, reduced patients’ access to life-saving care. These findings are consistent with the current body of literature evaluating provider well-being and staff turnover within substance use treatment and outreach services [22–32]. These studies identified that addressing these contextual factors in the workforce and providing more consideration to protect the well-being of healthcare staff through integrative social and workplace supports can improve addiction care across the entire continuum. The future implementation of broader co-worker supports and reflective supervision policies might also aid in this endeavor [26]. Findings from this study are similar, whereby many healthcare staff and provider participants showed interest in team-building activities to boost morale. Additional support through monthly meetings with managerial staff was also echoed to aid staff wellbeing within their role.

Interestingly, qualitative study findings also reported satisfaction resulting from the ability to provide flexible care plans. However, while some participants described the need for flexibility and individualized care to promote a patient-centered approach and strengthen patient engagement, others highlighted the need for more trauma-informed focus, employing clear rules and consistent care planning to maintain trust and equitable care among patients. Consistency and predictability are recognized as core elements of trauma-informed care, as they foster a sense of safety and trust for individuals who may have experienced chaotic or unpredictable environments [41]. Importantly, one of the most notable findings from this study was that perceptions regarding the value and implementation of “flexible care” appeared to differ across occupational roles. While many participants viewed flexibility and individualized decision-making as central to recovery-oriented care, others emphasized that excessive variability in rule enforcement or clinical decision-making could undermine trust, predictability, and psychological safety for both staff and patients. These differing perspectives were particularly evident in discussions surrounding substance use on the unit, patient-initiated discharge, and operational responses to situations perceived as high risk or unpredictable.

Qualitative findings suggested that staff whose roles involved direct implementation and enforcement of unit policies, particularly nurses and operations-focused staff, more frequently emphasized the importance of consistency, predictability, and clearly defined expectations in maintaining a safe and trauma-informed environment. In contrast, participants in other roles more often prioritized flexibility, discretion, and individualized approaches to care planning. This distinction represents an important operational tension within recovery-oriented inpatient substance use care and may reflect differences in role responsibilities, exposure to risk, and proximity to crisis response. Importantly, these findings suggest that flexibility itself may not be uniformly experienced by staff as inherently supportive or therapeutic. Rather, the perceived value of flexible care may depend heavily on how flexibility intersects with role expectations, safety concerns, workload demands, and accountability for operational decision-making. Further evaluation is needed to investigate whether differing role responsibilities impact staff views on providing person-centered care and the parameters in which their role flexibility allows for more individualized care while ensuring a level of equitable and consistent care for all.

The integration of traditional cultural healing practices and Indigenous healthcare was universally highlighted as a major strength and unique feature of the R2R model of care by healthcare staff. Incorporation of Indigenous perspectives at early stages of the design of R2R was intended to inform the development of a culturally safe environment, facilitate the implementation of Indigenous-based programming, and ensure the needs of Indigenous patients are met when receiving care through R2R. This was accomplished through direct partnership with Providence Health Care’s IWR team. The incorporation of Indigenous leadership with IWR guided the vision for Indigenous participation and ultimately the creation of the well-regarded Indigenous programming. Partnership with Indigenous leadership from the inception of the R2R model of care supported the development of a patient and Indigenous-focused model of care, inclusion of Indigenous health providers, and recognition of the beneficial role for incorporation of traditional healing practices, further adhering to the calls to action from the Truth and Reconciliation Commission of Canada [42, 43].

Finally, as R2R aims to restructure the substance use recovery care model of services and programming in BC, the results of this study underpin the need for continuous provider engagement and feedback across all stages of development and implementation. Healthcare staff involved in the provision of substance use care are continually evaluating and (re)defining what recovery-oriented care looks like within their own practice, specifically regarding the provision of trauma-informed and person-centered care. Addressing staff and provider-identified structural barriers that impede discharge planning and continuity of care would benefit people who use drugs and in turn boost the morale of those who work in the field of substance use care.

### Study limitations

As a single-site evaluation conducted within a specialized hospital-based withdrawal management program, the findings may not be generalizable to other healthcare settings or models of addiction care. Additionally, the qualitative findings are based on a small sample of healthcare workers and should be interpreted as providing in-depth contextual insights rather than representing the full range of staff experiences or perspectives. As this study was conducted within the first 12-months of operations, the study team was limited in observing the lasting impacts of R2R’s new ‘wraparound’ care model on healthcare staff experiences. This emphasizes the need for iterative service provision feedback from healthcare staff where quality improvement efforts can help evolve the delivery of care as well as identify areas of improvement closely associated with provider satisfaction. Beyond this, involvement of program directors in identifying potential participants may have introduced selection bias, particularly in interviews exploring experiences and job satisfaction within the program. Additionally, as it pertains to the qualitative component of the evaluation, the interviewer’s affiliation with the broader R2R program and research evaluation team may have influenced participant responses, particularly regarding perceptions of the program and workplace experiences. Finally, findings reflect perspectives within a medicalized substance use treatment setting and should be interpreted within this context.

## Conclusion

This mixed-methods study offered insights into the experiences of healthcare staff across diverse roles within the new R2R initiative. Participants generally expressed satisfaction with their roles and shared a common understanding of recovery-oriented care as strength-based, trauma-informed, and person-centered. Positive work experiences were linked to R2R’s integrated care model, inclusion of harm reduction and abstinence approaches, traditional healing practices, and the involvement of unique roles including peer support specialists and IWLs. Challenges included rigid responses to relapse and persistent staffing shortages, which contributed to burnout—issues consistent with broader trends in substance use care across North America. These findings offer practical insights for health systems developing or expanding hospital-based withdrawal management services, particularly regarding workforce engagement, culturally responsive care integration, and the balance between standardized protocols and individualized treatment approaches. As the model scales provincially, continued evaluation of staff experiences will be essential to understanding its long-term impact and adapting to evolving patient needs amid the toxic drug crisis.

## Supplementary Information

Below is the link to the electronic supplementary material.

Supplementary Material 1

Supplementary Material 2

## Data Availability

No datasets were generated or analysed during the current study.

## Declarations

The study protocol and related study materials was reviewed by the Chair of the University of British Columbia (UBC)-Providence Health Care Research Ethics Board and has been determined to be exempt from the requirements for researcher ethics approval both in accordance with UBC’s Human Research Policy LR9 and in accordance with the provisions of the Tri-Council Policy Statement: Ethical Conduct for Research Involving Humans, Article 2.5.

## Abbreviations

BC: British Columbia
BrAID: Brief Assessment at Intake and Discharge
IQR: Interquartile range
IWL: Indigenous Wellness Liaison
IWR: Indigenous Wellness and Reconciliation
RSA-R: Recovery Self-Assessment
R2R: Road to Recovery
SPH: St. Paul’s Hospital (Vancouver, Canada)
UBC: University of British Columbia
VCH: Vancouver Coastal Health
