# Supplementary material for: Implementing a new model of substance use care in British Columbia: healthcare staff experiences from the road to recovery initiative
Source: Addict Sci Clin Pract. 2026 Aug 6;21:84. doi: 10.1186/s13722-026-00705-y (PMC13737656; doi:10.1186/s13722-026-00705-y)
Supplement: Supplementary file 1 [file 13722_2026_705_MOESM2_ESM.pdf]

The Road to Recovery Initiative (R2R): an observational study of a novel model of substance use care in a Canadian setting

Baseline Qualitative Interview:

Health Care Provider Participants

Principal Investigator: **Seonaid Nolan MD, FRCPC**
Clinician Scientist, BC Centre on Substance Use (BCCSU)
Assistant Professor, Dept. Medicine, UBC
UBC Steven Diamond Professor in Addiction Care Innovation
Tel: 604-682-2344 ext. 62360
Seonaid.nolan@bccsu.ubc.ca

Road to Recovery Initiative (R2R) Health Care Provider Experiences

QUALITATIVE INTERVIEW COVER SHEET

COVER SHEET FOR PARTICIPANT INTERVIEWS REGARDING EXPERIENCES WORKING ON R2R (BASELINE)

Interviewer: ____

Date and Time: ____

Filename: ____

DIRECTIONS FOR INTERVIEWER:

ALL PARTICIPANTS:

INTERVIEW GUIDE FOR HEALTH CARE PROVIDERS REGARDING EXPERIENCES WORKING ON R2R (<u>BASELINE INTERVIEW</u>)

QUALITATIVE INTERVIEW GUIDE

INTERVIEW GUIDE FOR HEALTH CARE PROVIDER PARTICIPANTS REGARDING EXPERIENCES WORKING ON R2R SERVICES (BASELINE INTERVIEW)

DIRECTIONS

Thank you for agreeing to share your experiences as a health care provider working on the Road to Recovery Initiative (R2R) at Providence Health Care. As we discussed during the consent process, today you will be taking part in an approximately 30–60-minute interview about your background in substance use care and experiences with the R2R program. We want to understand your experiences as a health provider working with patients who use substances. We want to explore how the R2R is similar or different to previous experiences you have had working in substance use care. We are hoping to evaluate both positive and negative experiences and determine how we might work to improve the R2R to meet the needs of patients. As a health care provider, your experiences are important in both shaping and improving how we deliver care through the R2R. While we do ask for examples related to patient care, please do not provide any names or identifying information about individual patients. We will also invite you to participate in a future interview so that we can better understand your experiences with the R2R over time.

Please note that this interview will be audio recorded. Before we begin, please remember that everything that you share with us is confidential, and your information or identify will not be shared with anyone unless required by law. Also, please keep in mind that there are no right or wrong answers to these questions, and you do not have to answer any questions that you are not comfortable answering and can just say “skip” and we’ll move on. If there are any questions you don’t understand, please stop me and ask for clarification. The interview takes about 30-60 minutes. If you need a break, let me know and we can stop for a short rest before we finish the interview. Do you have any questions before I turn the recorder on?

IN-DEPTH INTERVIEW – START RECORDING

SECTION ONE: EXPERIENCES WORKING IN SUBSTANCE USE CARE	
“We are interested in understanding your previous experiences working in the field of addictions and substance use care…”	
Questions	***Probes***
Can you tell me a bit about your background as a health care provider?	▪ *What is your current role as a health care provider?*<br>▪ *How long have you worked in the field of substance use care?*<br>▪ *What type of training have you had in preparation for your role as a health care provider for patients who use substances?*<br>▪ *What previous work experience have you had in addictions care?*<br>▪ *Can you describe the types of places you have worked?*

	▪ *Have you worked in hospital-based settings? Detox? Community clinics?*<br>▪ *Have you worked in any bed-based treatment facilities (other names: residential treatment, rehabilitation centres)?*<br>▪ *What other roles have you had in addictions care?*
Can you tell me about more about your experiences with working in patients receiving treatment for substance use?	▪ *What types of patients with substance use disorders have you worked with? (Examples: all substance use? Alcohol? Opioid use?)*<br>▪ *How were your previous experiences working with patients who use substances?*<br>▪ *Can you describe any previous challenges you experienced working with patients receiving substance use care?*<br>▪ *Did you have any concerns about the way care was provided for patients in your previous work environments?*

SECTION TWO: EXPERIENCES WORKING ON R2R

"We are interested in understanding more about your experiences as a health care provider working on the R2R…."

Questions	***Probes***
Please describe your role on the R2R?	▪ *What is your role on the R2R?*<br>▪ *What types of services are you working on? R2R acute stabilization beds? Stabilization beds? Treatment beds?*<br>▪ *How long have you been a health care provider on the R2R?*
Can you describe the preparation for your role on the R2R?	▪ *Did you receive additional training for the R2R?*<br>▪ *Describe your orientation? (Examples: Types of sessions? How long was it?)*<br>▪ *Did you feel this was similar or different to previous orientations you've had to work in substance use care? Describe how.*<br>▪ *Do you feel the orientation was enough to prepare you for your role?*<br>▪ *What, if anything, could have been improved?*<br>▪ *Did you have an opportunity to provide feedback?*
Please describe your experiences working on the R2R as a health care provider.	▪ *Please describe your experiences working on the R2R?*<br>▪ *Can you describe a typical day on the R2R service and the tasks you are responsible for?*<br>▪ *In your role, what do you do for patients on the R2R? Do you believe this is helpful for patients? (Please elaborate with examples).*<br>▪ *What type of care do patients receive while they are on the R2R? Do you feel we are meeting their needs? If yes or no please provide further details?*

	▪ *Can you share any success stories or positive outcomes that you've witnessed while working with R2R? Are there any specific cases that stand out to you?*<br>▪ *Have your experiences thus far been similar or different to your previous experiences working in substance use care (please elaborate further)?*
Have there been any challenges or difficult experiences you've encountered working on the R2R service?	▪ *What challenges or obstacles have you encountered while working on this initiative, how did you and your team overcome them?*<br>▪ *How do you think these scenarios were handled?*<br>▪ *Do you find these obstacles/challenges were similar to those encountered in your previous work environments? Please elaborate.*<br>▪ *How do you think these challenges/difficult experiences impacted patient care?*<br>▪ *Have you felt or experienced stigma towards patients who use substances on the R2R? How do you and the R2R team address the stigma associated with addiction care?*<br>▪ *Can you describe any challenges related to resource allocation and funding that you've witnessed while operating within the R2R? Has this affected the quality of care?*<br>▪ *What obstacles do you encounter when engaging and retaining individuals in treatment, especially those who may be resistant or ambivalent about seeking help for their addiction?*<br>▪ *How has your experience been when patients initiate discharge? Are there protocols/methods to engaging patients to stay, or address their concerns?*<br>▪ *Have there been any legal or regulatory challenges that health care providers within the R2R must navigate, and how do these impact patient care and treatment options? (Example: patients on iOAT seeking bed-based treatment, experiences with patients on Extended Leave)*<br>▪ *What difficulties do you face in coordinating care and maintaining continuity of care for patients who may have complex needs, such as co-occurring mental health disorders? Have these been similar to your previous experiences?*<br>▪ *How do you manage challenges related to relapse among individuals in treatment, and what strategies are in place to provide ongoing support and encouragement? Has this been similar to your previous work experiences?*

	▪ *Are there challenges related to cultural competence and diversity in providing addiction care within the initiative, and how does the R2R address these issues?*
In your own words, describe how you perceive patients are experiencing the care provided to them on the R2R?	▪ *Do you think patients are satisfied with the care being provided? Please provide examples.*<br>▪ *From your perspective, how do you believe patients enrolled in R2R services perceive the quality of care they receive?*<br>▪ *What indicators or feedback mechanisms do you use to assess patient satisfaction?*<br>▪ *Can you share any patient testimonials or feedback that you've received, which provide insights into their overall experience with the care and support offered by the R2R Initiative?*<br>▪ ***How do health care providers within the R2R actively seek and incorporate patient input and preferences into their treatment plans and care decisions, and what impact does this patient-centered approach have on the patient experience?***<br>▪ *What strategies or initiatives does the R2R Initiative employ to ensure that patients feel heard, respected, and empowered in their addiction recovery journey, and how do these strategies contribute to positive patient experiences?*
Please describe how your role as a health care provider on the R2R has impacted your job satisfaction.<br><br>(*These questions can help gauge health care providers' satisfaction and well-being while working on the Road to Recovery Initiative, shedding light on their personal and professional experiences within the program)*	▪ *How has your experience working on the R2R impacted your overall job satisfaction and career fulfillment?*<br>▪ *What aspects of the initiative's work environment, team dynamics, or organizational support contribute to your job satisfaction? Please describe both positive and negative experiences.*<br>▪ *Have you had opportunities for professional development or training related to addiction care while working on the initiative, and if so, how has this influenced your job satisfaction and competence?*<br>▪ *Can you describe any professional challenges or stressors you've encountered in your role within the R2R, and how has the organization supported you in managing them?*<br>▪ *What feedback mechanisms or avenues for communication exist within the initiative to allow health care providers to voice concerns, share ideas, or offer suggestions for improvement?*<br>▪ *Have you had opportunities to collaborate with other professionals or disciplines within the initiative, and if so, how has this interdisciplinary approach impacted your job satisfaction and patient care?*

	▪ *Can you share any personal or professional growth experiences that you've gained through your involvement with the initiative, and how have these contributed to your job satisfaction and skill development?*<br>▪ *In your opinion, what sets the R2R apart from other addiction care services, and how does this contribute to a more/less satisfying work experience?*

SECTION THREE: HOW TO IMPROVE/CHANGE THE R2R SERVICE TO BETTER MEET THE NEEDS OF PATIENTS WHO USE SUBSTANCES

"Now we want to learn more about how to improve the R2R services to better meet the needs of patients who use substances, we are interested to learn how you think the service could be improved."

<u>**Questions**</u>	<u>**Probes**</u>
What ways could the R2R be improved to better meet the needs for patients who use substances?	▪ *Please describe how you feel the R2R could be improved?*<br>▪ *Are there specific challenges or areas of improvement that you have identified in terms of enhancing the patient experience within the R2R Initiative?*<br>▪ *In your experience, what are some specific areas of the R2R that you believe could benefit from modifications to better meet the needs of individuals seeking addiction care?*<br>▪ *Can you identify any gaps in the current services or treatment modalities offered by the R2R Initiative, and do you have suggestions for filling these gaps to improve patient outcomes?*<br>▪ *Are there any technological advancements or innovative tools that you believe could be integrated into the R2R Initiative to streamline operations, enhance communication, or improve patient engagement?*<br>▪ *What strategies or interventions do you think could be implemented to further reduce the stigma associated with addiction care and encourage more individuals to seek help through the R2R Initiative?*<br>▪ *In your opinion, how can the R2R Initiative strengthen its collaboration with other community organizations or health care providers to create a more comprehensive and seamless addiction care network?*<br>▪ *Are there specific training or education opportunities that you believe would benefit health care providers within the R2R Initiative, helping them better serve their patients and stay updated on the latest addiction care practices?*<br>▪ *How can the R2R Initiative better support health care providers in managing their own well-being and coping with the emotional demands of addiction care to prevent burnout and enhance job satisfaction?*

	▪ *Do you have any suggestions for collecting and utilizing patient feedback more effectively to inform continuous improvement efforts within the R2R Initiative?*
Based on your experiences, what do you feel are the most important changes that could be made to improve Road to Recovery program, particularly for (Indigenous or minority) women (of colour) and women-identified people? What changes would you like to see?	
Is there anything that we haven't discussed that you feel is important for us to know about?	
