# Supplementary material for: Implementing a new model of substance use care in British Columbia: healthcare staff experiences from the road to recovery initiative
Source: Addict Sci Clin Pract. 2026 Aug 6;21:84. doi: 10.1186/s13722-026-00705-y (PMC13737656; doi:10.1186/s13722-026-00705-y)
Supplement: Supplementary file 2 [file 13722_2026_705_MOESM1_ESM.docx]

# Road to Recovery - Health Services Evaluation

# Staff Questionnaire

We are interested to hear about your experiences working as part of the Road to Recovery (R2R) Initiative! The purpose of this survey is to solicit feedback from team members, including: addiction care providers, project coordinators, clinical and research staff, or administrators involved in the R2R service development or daily service provision. We would like to learn about your experiences working to support this new model of care, identify challenges that have affected patient care and work-flow, and ultimately generate suggestions for how to effectively improve service delivery for this important patient population.

Anyone who has contributed to: (1) the development of R2R, or (2) provides clinical care for patients through any R2R service is eligible to take part in this survey, which will involve completion of at least one questionnaire estimated to take 30-45 minutes of your time. It is completely up to you if you would like to participate. Whether or not you decide not to participate, your decision will have no negative impact on your role as a team member with the R2R. If you decide to participate, it is important to understand that there is no financial compensation. The results of this survey and other follow-up survey opportunities will be used to help improve the current system of addiction care provided through R2R and inform its scale up and implementation in other areas across the province.

**Date: _____________________**

**Would you like to be contacted 12 months from now to participate in our follow-up survey?**

| - Yes | - No |
| --- | --- |

- ***If yes: Please provide your email and phone number***
  - **Email: ________________**
  - **Phone: ________________**

**SECTION A: STAFF DEMOGRAPHICS**

A1 What gender do you identify with ***(check all that apply)***:

- Man
- Woman
- Non-binary
- Intersex
- Transgender
- Prefer to self-describe:____________
- Two-spirit
- Prefer not to say

A2 What was your biological sex at birth?

- Male
- Female
- Other: ______

A3 What sexuality do you identify with?

- Straight/heterosexual
- Gay/lesbian/homosexual
- Asexual
- Bisexual
- Two-spirit
- Prefer to self-describe:____________________
- Queer
- Pansexual
- Prefer not to say

A4 How old are you?

_____ years old

A5 Do you identify as an Indigenous person?

- Yes
- No
- Prefer not to answer

a) If yes, do you identify as First Nations, Metis or Inuit:

- First Nations
- First Nations and Métis
- First Nations and Inuit
- First Nations and Métis and Inuit
- Inuit
- Métis
- Inuit and Métis
- No response
- Unknown
- Other (please specify:______)

A6 What do you identify your race or ethnicity as? ***(Check all that apply)***

|  | White |
| --- | --- |
|  | South Asian (e.g., Indian, Pakistani) |
|  | Chinese |
|  | Other Asian (e.g., Vietnamese, Japanese) |
|  | Latin American |
|  | Middle Eastern |
|  | Black African |
|  | Black Caribbean |
|  | Other, specify:__________________________________ |

A6 What is your role as part of the R2R team?

- Doctor
- Peer Support Worker
- Hospital Administrator
- Nurse
- Pharmacist
- Researcher
- Social Worker
- Project Manager/Coordinator
- Allied Health (OT/Physio)
- Architect/construction
- Indigenous Wellness Reconciliation team
- Unit Clerk
- Security
- Care Aide
- Other, please specify: _________

A7 How long have you been in your R2R role? (In MONTHS)

- <3 months
- 3-6 months
- 6-12 months
- >12 months

A8 Have you worked in the addiction, substance use field previously?

| - Yes | - No |
| --- | --- |

a) *If yes,* how long have you worked in substance use care?
   - <1 year
   - 1-5 years
   - 5-10 years
   - >10 years
b) *If yes,* what settings have you worked in?
   - Withdrawal management/detox
   - Hospital based care
   - Outpatient clinic

A9 How long have you worked in your role (length of time you’ve been a doctor, nurse, social worker, etc.)

- <1 year
- 1-5 years
- 5-10 years
- >10 years

**B: CLINICAL DOCUMENTATION**

B1 I feel that the BRAID form collects useful clinical information

| - Yes | - No | - N/A |  |
| --- | --- | --- | --- |

a) If no, please explain why in a few words: ________________________________________________________________________________________________________________________________________________________________________________________________________________________

B2 I have received enough training to administer the BRAID form confidently

- Strongly disagree
- Disagree
- Neutral
- Agree
- Strongly agree

B3 I feel that I have enough time to administer the BRAID form

| - Yes **(IF YES, SKIP TO QUESTION B4)** | - No |
| --- | --- |

a) If no: What could help support you in collecting this data? **(Check all that apply)**
   - Additional trainees
   - Reminders
   - Contingency management incentives
   - Other: ________

B4 I feel that there are not enough questions on the BRAID form to accurately document clinical information

- Strongly disagree
- Disagree
- Neutral
- Agree
- Strongly agree

B5 I feel that there are too many questions on the BRAID form

- Strongly disagree
- Disagree
- Neutral
- Agree
- Strongly agree

**C: WORKFLOW EFFICIENCY**

C1 I feel that rounds are efficient

- Strongly disagree
- Disagree
- Agree
- Strongly agree
- N/A

C2 I feel that patient intake is efficient

- Strongly disagree
- Disagree
- Agree
- Strongly agree
- N/A

C3 I feel that the admissions processes is efficient

- Strongly disagree
- Disagree
- Agree
- Strongly agree
- N/A

C4 I feel that the processes of medication administration is efficient

- Strongly disagree
- Disagree
- Agree
- Strongly agree
- N/A

C5 I feel that the processes of connecting patient with community resources is efficient

- Strongly disagree
- Disagree
- Agree
- Strongly agree
- N/A

C6 I feel that the process for follow-ups is efficient

- Strongly disagree
- Disagree
- Agree
- Strongly agree
- N/A

*The below questions are specific to certain roles. Please fill out the section for the role that you are in. Not all roles will have a section, if you do not see your role here then please skip to Section D.*

**A: Nursing**

CA1 The following aspect(s) of my workflow create the *biggest hurdles* to efficiency in my overall workflow ***(check all that apply)***

- Shift report
- Check in
- 8am medication distribution
- Smoke break
- Breakfast
- Hourly pain medication distribution
- Hourly assessments
- Other: __________

CA2 The following aspect(s) of my workflow are the *most efficient* in my overall workflow ***(check all that apply)***

- Shift report
- Check in
- 8am medication distribution
- Smoke break
- Breakfast
- Hourly pain medication distribution
- Hourly assessments
- Other: __________

CA3 The following aspects of the intake process create the *biggest hurdles* to intake efficiency ***(check all that apply)***

- Filling out the BRAID form
- Vitals
- Waiting for patient to finish showering
- Full assessment
- Intake patients coming early
- Previous patient has not been discharged yet
- Patients experiencing distress can activate other patients
- Other:________

CA4 The following aspects of the intake process are the *most efficient* ***(check all that apply)***

- Filling out the BRAID form
- Vitals
- Getting patient setup in the unit (showering, sorting through belongings)
- Full assessment
- Other:________

CA5 Please outline any suggestions you have for improving the workflow efficiency in this role that have not yet been captured in this survey __________________________________________________________________________________________________________________________________________________________________________________________________________________________________________

**B: Social work**

CB1 The following aspect(s) of my workflow create the *biggest hurdles* to efficiency in my overall workflow

- Keeping track of beds
- Receiving referrals from our unit
- Determining which beds are suitable for who
- Filling out applications
- Helping with appointment creation
- Receiving referrals from the urban health unit
- Collaborating with VCH stabilization to swap beds
- Discharge planning
- Supporting long-term stay patients
- Other: ________

CB2 The following aspect(s) of my workflow are the *most efficient* in my overall workflow

- Keeping track of beds
- Receiving referrals from our unit
- Determining which beds are suitable for who
- Filling out applications
- Helping with appointment creation
- Receiving referrals from the urban health unit
- Collaborating with VCH stabilization to swap beds
- Discharge planning
- Supporting long-term stay patients
- Other: ________

CB3 Please outline any suggestions you have for improving the workflow efficiency in this role that have not yet been captured in this survey

__________________________________________________________________________________________________________________________________________________________________________________________________________________________________________

**C: Pharmacy**

CC1 The following aspect(s) of my workflow create the *biggest hurdles* to efficiency in my overall workflow ***(check all that apply)***

- Consulting with patients after referral from social work
- Liaising with physicians on treatment and feasibility
- Induction of OAT
- Intake
- Admission
- Creating medication requisitions
- Managing medications and conditions
- Disposition planning
- Discharge prescriptions
- Connecting with community pharmacy
- Sending scripts to right pharmacy
- Other: _________

CC2 The following aspect(s) of my workflow are the *most efficient* in my overall workflow ***(check all that apply)***

- Consulting with patients after referral from social work
- Liaising with physicians on treatment and feasibility
- Induction of OAT
- Intake
- Admission
- Creating medication requisitions
- Managing medications and conditions
- Disposition planning
- Discharge prescriptions
- Connecting with community pharmacy
- Sending scripts to right pharmacy
- Other: _________

CC3 Please outline any suggestions you have for improving the workflow efficiency in this role that have not yet been captured in this survey __________________________________________________________________________________________________________________________________________________________________________________________________________________________________________

**D: TEAM DYNAMICS AND TEAM CULTURE**

D1 I feel part of a team in working towards shared goals

- Strongly disagree
- Disagree
- Agree
- Strongly agree

D2 I receive assistance from coworkers when necessary

- Strongly disagree
- Disagree
- Agree
- Strongly agree

D3 I would be interested in team building exercises

| - Yes | - No |
| --- | --- |

D4 I know where to go within the organization to obtain information that I need

- Strongly disagree
- Disagree
- Agree
- Strongly agree

D5 I feel that my feedback is heard and appropriate actions are taken to address my concerns

- Strongly disagree
- Disagree
- Agree
- Strongly agree
- N/A

a) If any answer except N/A: Please provide an example of a time you provided feedback and actions were/were not taken to address it ________________________________________________________________________________________________________________________________________________________________________________________________________________________

**E: JOB SATISFACTION**

Please note that responses collected in this section for workplace improvements are suggestions only. Any changes to the workplace are subject to operational considerations (ex. budget, space, availability of resources).

E1 My initial training provided by the hospital was sufficient

- Strongly disagree
- Disagree
- Agree
- Strongly agree

E2 I am satisfied with my total working hours

- Strongly disagree
- Disagree
- Agree
- Strongly agree

E3 My physical working conditions (lighting, ventilation, ergonomics, etc.) are good

- Strongly disagree
- Disagree
- Agree
- Strongly agree

E4 The following additional supports would help me do my job

- Monthly meetings with RNs and managerial staff (CNE, CNL)
- More check ins with managerial staff
- Formal training specific to the unit
- Clerical support
- Hiring more staff in this role
- Adequate Office Space
- Additional hiring for addiction medicine educators hospital-wide (not just R2R)
- Regular huddles for refreshing standard practice — i.e. CIWA scoring and subsequent medication dispensation
- Mentoring/additional training related to ensuring a culturally safe environment for Indigenous patients
- Other: __________

E5 I feel that the R2R program supports my own health and wellness

- Strongly disagree
- Disagree
- Agree
- Strongly agree

E6 The following additional supports would help me with my personal wellness ***(check all that apply)***

- Protected break times
- Additional opportunities for socializing during or after work
- Team events to increase team spirit
- Additional hiring for this role
- Other: ___________

E7 Overall, I feel satisfied with my job

- Strongly disagree
- Disagree
- Agree
- Strongly agree

**F: PROGRAM VIEWS**

F1 The mission and purpose of my organization make me feel that my job is important

- Strongly disagree
- Disagree
- Agree
- Strongly agree

F2 Staff make a concerted effort to welcome people in recovery and help them to feel comfortable in this program

- Strongly disagree
- Disagree
- Neutral
- Agree
- Strongly agree
- Don’t know
- N/A

F3 Staff encourage program participants to have hope and high expectations for their recovery

- Strongly disagree
- Disagree
- Neutral
- Agree
- Strongly agree
- Don’t know
- N/A

F4 Staff listen to and respect the decisions that program participants make about their treatment and care

- Strongly disagree
- Disagree
- Neutral
- Agree
- Strongly agree
- Don’t know
- N/A

F5 I feel that the program was designed with patient preferences in mind

- Strongly disagree
- Disagree
- Neutral
- Agree
- Strongly agree

F6 Staff are committed to creating a culturally safe environment for patients and their families.

- Strongly disagree
- Disagree
- Neutral
- Agree
- Strongly agree
- Don’t know
- N/A

F7 I feel there could have been better approaches to the design and evaluation of the R2R services

| - Yes | - No **(IF NO, SKIP TO QUESTION F7)** |
| --- | --- |

a) If yes, please explain:

_________________________________________________________________________________________________________________________________________________________________________________________________________________________________

F8 Do you feel all stakeholders were involved in the decision making process when developing and assessing the R2R?

| - Yes **(IF YES, SKIP TO SECTION G)** | - No |
| --- | --- |

a) If no, please select which stakeholders you feel were not adequately involved ***(check all that apply)***:
   - Indigenous
   - Pregnant
   - Youth populations
   - Other: _______

**G: VIEWS ON EFFECTIVE CARE**

G1 I feel that the care being provided at the R2R is ***(check all that apply):***

- Safe
- Trauma-informed
- Patient centered
- Culturally and spiritually sensitive
- None of the above

G2 I feel that stigma/negative attitudes towards substance use *is present* among R2RI service staff/patients

- Strongly disagree
- Disagree
- Agree
- Strongly agre**e**

G3 What is different about R2R compared to other experiences in substance use care? ***(check all that apply):***

- Access to a wider number of treatments
- More privacy for patients
- More patient centered
- Better connections to community and subspecialty care
- More opportunities for psychosocial treatment
- Certain activities are more available (ex. Yoga)
- Recovery housing provided
- Transitional care beds provided
- Faster access to care
- Care is more well-rounded
- Access to employment/vocational services
- None of the above
- Other, please specify: ______
